# Exercogs®: Technology Solution Applied in Dementia Prevention

**DOI:** 10.1192/j.eurpsy.2025.1748

**Published:** 2025-08-26

**Authors:** C. Pombo, P. Varandas, C. Gameiro, B. Lopes, B. Freitas, S. Rosado, A. Bernardino, H. Cardoso

**Affiliations:** 1 Aging and Dementia; 2 Clinical Management, Sisters Hospitallers; 3 Institute for Systems and Robotics, Instituto Superior Técnico (University of Lisbon), Lisbon, Portugal

## Abstract

**Introduction:**

Recent research highlights the importance of technological solutions in delaying cognitive decline and improving life quality for at-risk individuals, emphasizing the need for innovative, tech-based approaches in dementia prevention (Johnson et al.,2023; Smith et al.,2023). Exercogs® is an innovative tool designed to tackle the global challenge of dementia by addressing modifiable risk factors (Livingston et al.,2020). Developed through a clinic-academia partnership, it integrates physical exercise, cognitive skills, and social interaction into a single activity for dementia prevention programs.

**Objectives:**

This scientific study aims to: 1) Creating and validating 4 Exercogs® (using gamification) for an augmented reality platform; 2) Validating a dementia prevention program that generates health benefits using Exercogs®.

**Methods:**

1. **Research and Planning:** assessment of seniors’ needs; survey of market solutions;
2. **Ideation and Concept Development:** definition of therapeutic objectives; selection of stimulus types; idealization of game scenarios, cognitive, and motor areas;
3. **Design and Prototyping:** programming video games; implementation of gamification techniques;
4. **Testing and Evaluation:** testing the prototypes and interaction mechanics with a group of users; usability testing.;
5. **Scientific Validation:** experimental study with pre and post-test assessment, with a sample of 204 subjects aged ≥ 65 years old.
6. **Maintenance and Improvement:** the solution is being used in pilot studies in different institutions in Portugal for evaluation and continuous improvement.

**Results:**

Exercogs® consists of 4 games that target key areas of healthy and pathological ageing, focusing on cognitive (attention, memory, executive functions), physical (mobility, coordination, balance) and social (general social skills) domains. Each game adapts to users’ abilities with different difficulty levels and is designed for group play to enhance social interaction, crucial for mental health. Utilizing gamification and augmented reality for engagement, scientific validation showed significant improvements in cognitive, affective, social, functional domains, and quality of life, with marked statistical significance in all areas assessed.

**Image 1:**

**Figure:**
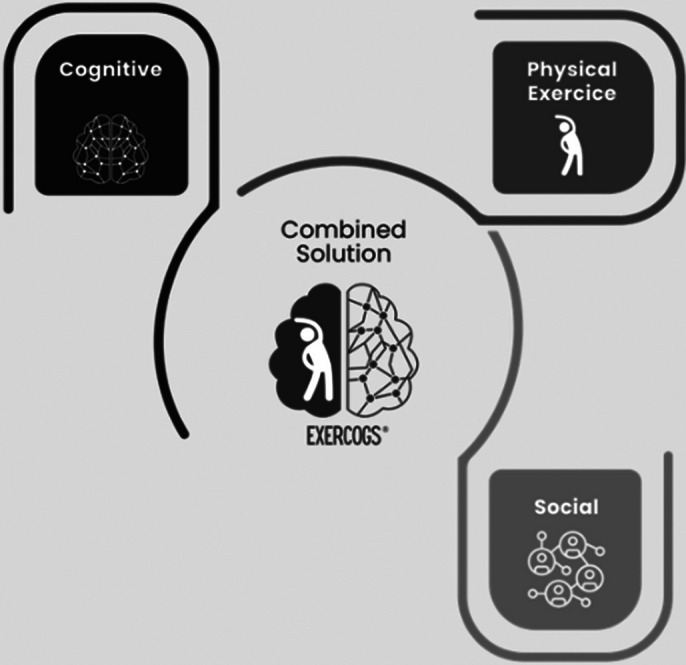

**Image 2:**

**Figure:**
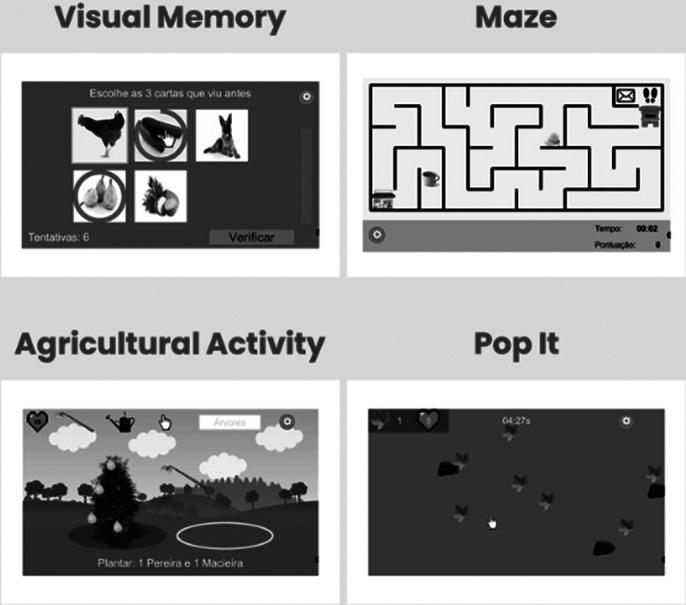

**Conclusions:**

Notable health benefits were observed among users, particularly in cognitive function and quality of life. These findings indicate the potential effectiveness of Exercogs® in dementia prevention programs. The alliance between the clinic and academia is crucial for solving the challenges of longevity and creating technological solutions that respond to new health needs. The use of technologies in health intervention generates high levels of adherence and motivation among older adults, as well as among health professionals. Exercogs® are a promising technological solution that uses gamification with clinical support to prevent dementia!

**Disclosure of Interest:**

None Declared

